# SNRK facilitates cardiac repair associated with nonischemic fibrosis: regulating transforming growth factor-beta1 levels in atrial cardiomyocytes

**DOI:** 10.4103/regenmed.regenmed-d-25-00009

**Published:** 2025-06

**Authors:** Karthikeyan Thirugnanam, Farhan Rizvi, Arshad Jahangir, Peter Homar, Fathima Shabnam, Sean P. Palecek, Suresh N. Kumar, Amy Pan, Xiaowen Bai, Hidekazu Sekine, Ramani Ramchandran

**Affiliations:** 1 Department of Pediatrics, Division of Neonatology, Developmental Vascular Biology Program, Children’s Research Institute (CRI), Milwaukee, WI, USA; 2 Aurora Cardiovascular and Thoracic Services, Advocate Health, Milwaukee, WI, USA; 3 Department of Chemical and Biological Engineering, University of Wisconsin-Madison, Madison, WI, USA; 4 Department of Pathology, Division of Pediatric Pathology, Medical College of Wisconsin, Milwaukee, WI, USA; 5 Department of Pediatrics, Division of Bioinformatics and Quantitative Child Health, Milwaukee, WI, USA; 6 Department of Cell Biology, Neurobiology and Anatomy, Medical College of Wisconsin, Milwaukee, WI, USA; 7 Institute of Advanced Biomedical Engineering and Science, Tokyo Women’s Medical University, Tokyo, Japan

**Keywords:** cardiac repair, cardiomyocyte, heart failure, HL-1, paracrine, regeneration, regenerative medicine, transforming growth factor-β1

## Abstract

Heart failure is a pressing clinical condition that is expected to increase as our population ages and thus requires better treatment options. Identifying the precise mechanisms that underlie fibrosis and inflammation, two key features associated with cardiac repair and regeneration during ischemic and nonischemic heart failure, is likely to provide effective strategies for the clinical intervention of heart failure. This study investigated a metabolic serine threonine kinase gene, sucrose nonfermenting-related kinase (*SNRK*), which we previously reported to control cardiac metabolism and function. Conditional knockout of *Snrk* in mouse cardiomyocytes (*Snrk* cmcKO) leads to deleterious fibrosis, inflammation, and, subsequently, heart failure. The precise mechanism underlying cardiomyocyte SNRK-driven repression of deleterious cardiac fibrosis in nonischemic heart failure-mediated cardiac repair and regeneration is not known. Here, using mouse, rat, and human tissues, we demonstrated that SNRK expression is increased in the atrial chamber, especially in left atrial cardiomyocytes. Using a nonischemic heart failure mouse model, we showed that fibrosis in the atria, particularly the left atria, is associated with cardiac functional decline. To elucidate the mechanistic pathway responsible for the SNRK-mediated repression of cardiac fibrosis, we focused on the profibrotic protein transforming growth factor-β1. Transforming growth factor-β1 levels in *Snrk* siRNA-knockdown HL-1 adult immortalized mouse atrial cells were higher compared with control siRNA-knockdown HL-1 cells. Coculture of HL-1 cardiomyocytes (−/+ *Snrk*) with cardiac fibroblasts *in vitro* revealed that SNRK represses transforming growth factor-β1 signaling (Smad2/3) in cardiac fibroblasts and cardiac fibroblast activation (alpha-smooth muscle actin marker). We conclude that under nonischemic heart failure conditions, increased SNRK expression in the atria is associated with a cardioprotective mechanism by controlling the release of the profibrotic transforming growth factor-β1 factor. These studies illuminate a potential deleterious fibrosis pathway for intervention during cardiac repair and regeneration in nonischemic heart failure.

## Graphical Abstract

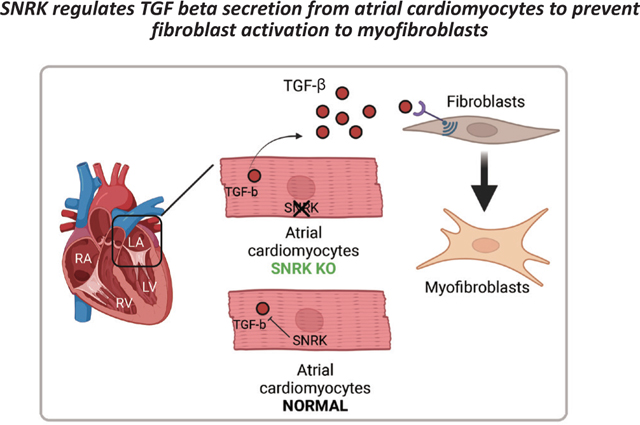

## Introduction

Approximately one in 4 persons will develop heart failure (HF) in their lifetime in the United States, and the prevalence is expected to increase with age.^1^ Thus, clinical interventions for HF are urgently needed. HF is associated with cardiac remodeling, a postinjury cardiac repair process in which inflammation and fibrosis processes play key roles in restoring tissue homeostasis.^2^ Both of these processes are reversible in preclinical and clinical settings, which forms the underlying basis for HF treatment options.^3,4^ Cardiac inflammation and fibrosis and postinjury cardiac repair processes are associated with ischemic and nonischemic HF.^2^ For example, in ischemic HF, inflammation precedes fibrosis, whereas in nonischemic HF, inflammation and fibrosis cooccur.^2^ Identifying targets that participate in both cardiac inflammation and fibrosis pathways and the underlying mechanisms is important for the development of target-based therapies associated with cardiac repair and regeneration to prevent HF.

We and others have investigated the role of one such target, sucrose nonfermenting-related kinase (SNRK), an AMPK family member in the cardiac system.^5–8^ *Snrk* is essential for cardiac metabolism and function, and *Snrk* cardiac conditional (*MYH6-CRE*) knockout (*Snrk* cmcKO) adult mice die at 9 months.^6^ At 4–5 months, when we subjected *Snrk* cmcKO mice to chronic infusion of angiotensin II (Ang II), a mouse model of ischemic HF, these mice died from HF within 2 weeks postinfusion compared with their control littermates.^7^ In contrast, 4–5-month-old endothelial-specific (*TIE2-CRE*) *Snrk* knockout (*Snrk* ecKO) mice subjected to Ang II infusion did not succumb. Others have shown that *Snrk* cardiac-specific overexpression in mice improved cardiac mitochondrial efficiency and decreased mitochondrial uncoupling in cardiomyocytes.^8^ Collectively, these data suggest that SNRK functions in cardiomyocytes (CMs) in a cell-autonomous and cardioprotective manner.

We evaluated cardiac fibrosis in *Snrk* cmcKO and *Snrk* ecKO hearts and found that in the basal state, *Snrk* cmcKO hearts presented enhanced fibrosis, whereas *Snrk* ecKO hearts did not.^7^ We also assessed the inflammatory pathway in *Snrk*-knockdown atrial HL-1 CMs in culture and reported that SNRK attenuates nuclear factor-κB-mediated inflammatory signaling.^7^ However, the underlying mechanisms associated with the cardiomyocyte SNRK-mediated repression of cardiac fibrosis are not known, which was the objective of this study. Here, we investigated the underlying mechanisms that contribute to the SNRK cardiomyocyte-driven repression of cardiac repair fibrosis process in nonischemic HF. In particular, we investigated the involvement of transforming growth factor-beta 1 (TGF-β1), a multifunctional cytokine that plays an important role in cardiac fibrosis, wound, and tissue repair,^9–11^ and its relationship with SNRK.

## Methods

### Animals

#### Mice:

The mice were housed at the Medical College of Wisconsin Biological Resource Center, and all experiments were performed following an approved Institutional Animal Care and Use Committee (IACUC) animal protocol 1022 (August 20, 2018). *Snrk cmcKO* (cardiomyocyte knockout) adult mice were generated from *MYH6*^CRE^*-*positive *Snrk*^LoxP^/wild-type (WT) males mated with *Snrk*^*LoxP/LoxP*^ females. The genotyping and characterization information for these alleles are available from our previous publication.^6^ The ages of the mice in the wild-type and *Snrk* cmcKO groups ranged from 3 months, 5 days (youngest), to 4 months, 18 days (oldest). The *in vivo* experiments included the following conditions: *Snrk* WT, *Snrk cmcKO, Snrk* WT with Ang II, and *Snrk cmcKO* with Ang II. Ang II osmotic pump infusion studies at a dose of 1000 ng/kg/minute were performed as described previously (see Supplemental Material). The experimental animals for the data points were all males in the study and *n* = 3 for each condition. We initially began the experiment with 5–7 mice per group, but due to mortality associated with Ang II infusion, we had only 3 remaining at day 14 for analysis.

#### Rats:

Sprague–Dawley rats (aged 8 weeks; male) were obtained from Japan SLC, Inc. In this study, we used 8-week-old male Sprague–Dawley rats (body weight 300–320 g). These rats were wild-type animals with no genetic manipulation or specific genetic background. They had not been subjected to any drug treatment or other experiments prior to the start of the study and were confirmed to be healthy individuals with no abnormalities in their immune system. Furthermore, they were maintained under specific pathogen-free (SPF) conditions with appropriate environmental hygiene management.

All animal experiments were performed following the guidelines of the Ethical Committee for Animal Experimentation of Tokyo Women’s Medical University and in compliance with the laws and regulations on the use of animals in biological research, and this study was reported in accordance with the ARRIVE 2.0 guidelines (Animal Research: Reporting of *In Vivo* Experiments).^12^ All experimental protocols were approved on March 30, 2023, by the Animal Experimentation Committee of Tokyo Women’s Medical University under approval No. AE23–104. The rats were housed in individual cages with free access to food and water under a 12-hour light/dark cycle and were kept at a constant room temperature and humidity. The animals were euthanized by exsanguination under 5% isoflurane, in accordance with the American Veterinary Medical Association (AVMA) euthanasia guidelines.

### Angiotensin infusion studies

#### Rats

This study was carried out using hearts from 12-week-old rats weighing 300–350 g, purchased at 8 weeks and weighing 300–320 g. Rats were anesthetized and subcutaneously implanted with an osmotic mini-pump (ALZET Osmotic Pumps 2ML4, Cupertino, CA, USA) that released angiotensin II (KareBay Biochem, Monmouth Junction, NJ, USA) at a constant rate of approximately 1.4 mg/kg per day for 4 weeks to create a model of cardiac inflammation and fibrosis.

#### Mice

To simulate cardiac stress, osmotic pumps that secrete a steady flow of angiotensin II were used. The pumps were implanted at 4 months of age, and transthoracic ECHO analysis was performed as described previously^7^ immediately prior to implantation, followed by analysis once per week for a total of 2–4 weeks. At the end of the experiment, the mice were euthanized via 30–70% CO_2_ per minute of chamber volume and cervical dislocation. Details of the osmotic pump installation are available in the supplemental material section.

#### Human studies

The study was approved by the Aurora Institutional Review Board and conforms to the Health Insurance Portability and Accountability Act of 1996. The study was conducted after receiving informed consent from the patients. All nonheart failure samples were obtained from the National Disease Research Interchange, Philadelphia, PA. All the HF samples were obtained from heart transplant patients, and all of them had a reduced ejection fraction (EF<45). Atrial and ventricular tissues were dissected and lysed in RIPA buffer for western blot analysis. Details of the patient information are available in Additional Figure 1G.

### Immunohistochemistry

#### Macrophage staining

Formalin-fixed hearts collected from *Snrk* WT and *Snrk cmcKO* mice treated independently with vehicle or Ang II (1000 ng/kg/minute) were prepared as described previously.^7^ Briefly, to assess macrophage infiltration in the heart, an anti-mouse/human Mac2 (Galectin-3) antibody (Cat# CL8942AP; Cedarlane, Burlington, ON, Canada) was used. Pretreatment with target retrieval solution (S1699, Dako, Santa Clara, CA, USA) was performed by heating (95°C) over a period of 40 minutes, followed by cooling at room temperature for 15 minutes. Tissue slices were incubated overnight at 4°C, and stained sections were viewed via light microscopy (Carl Zeiss AG, Oberkochen, Germany). All analyses of histological data quantification were performed via scanned images that were imported into Visiopharm software (Visiopharm A/S, Hørsholm, Denmark) via the imager module; 10 × 20 region-of-interest images were extracted from the left, center, and right regions of the heart. All original images were processed with this preset threshold and linear Bayesian classification to generate the processed image.

#### Fibrosis studies

Picrosirius red (Abcam, Cambridge, MA, USA) staining was performed to determine the extent of fibrosis in the heart sections. The amount of fibrosis was quantified via a software-assisted (Visiopharm A/S, Hørsholm, Denmark), unbiased microimage quantification method. Image analysis was blinded without the analyst’s knowledge of the groups and treatments. The scanned images were imported into Visiopharm software (Hørsholm, Denmark), and the region of interest was marked in each of the heart’s left, center, and right ventricular walls. Similarly, the left and right atrium wall areas were also marked for analysis. All original images were processed at 20× magnification with a preset threshold to estimate the fibrotic region (red stain), and linear Bayesian classification was performed to generate the processed image. The perivascular fibrotic region was identified using the lumen in the center and eliminated by a postprocessing filter. The total interstitial collagen-positive area per region of interest was measured in micrometers and represented as a percentage of the total tissue area.

#### Alpha smooth muscle actin staining

Briefly, formalin-fixed hearts collected from WT and *Snrk* cmcKO mice treated independently with vehicle or Ang II were dehydrated, embedded in paraffin, and sectioned at 5 μm. The slides were warmed at 60°C for 1 hour, dewaxed in xylene, and rehydrated. To assess alpha-smooth muscle actin expression, an anti-alpha smooth muscle actin antibody (1:200 dilution, Cat# 5694, Abcam) was used, followed by an anti-rabbit secondary antibody (Cat# 150093, Abcam) for 1 hour at room temperature. Pretreatment with target retrieval solution (S1699, Dako) was performed by heating (95°C) over a period of 40 minutes and then cooling at room temperature for 15 minutes. The tissue slices were incubated overnight at 4°C, and the stained sections were viewed using light microscopy (Carl Zeiss AG). All analyses of histological data quantification were performed with scanned images that were imported into Visiopharm software (Hørsholm, Denmark) using the imager module; 10 × 20 region-of-interest images were extracted from the left, center, and right regions of the heart. All original images were processed with this preset threshold and linear Bayesian classification to generate the processed image.

### Immunofluorescence studies

#### Rat tissue immunofluorescence studies

For cross-sectional observation, rat hearts were fixed in 4% paraformaldehyde and processed into 7 µm-thick paraffin-embedded sections. For detection of SNRK, deparaffinized sections were incubated with a 1:100 dilution of anti-SNRK rabbit polyclonal antibody (Cat# GTX111380, Genetex, Irvine, CA, USA). For detection of cardiomyocytes, the sections were incubated with a 1:100 dilution of anti-cardiac troponin T rabbit polyclonal antibody (Abcam), and for detection of the TGFβ protein, the sections were incubated with a 1:100 dilution of anti-TGFβ rabbit polyclonal antibody (Cat# GTX110630, Genetex) at 4°C overnight. The sections were then incubated with a 1:200 dilution of Opal Polymer anti-Rabbit HRP (Cat# ARR1001KT; Akoya Biosciences, Marlborough, MA, USA) for 1 hour at room temperature. Both the primary and secondary antibodies were diluted in 1× PBS. The signals were generated using Opal Fluorophore (Opal520, 570, Akoya Biosciences), and the nuclei were counterstained with Spectral DAPI (Akoya Biosciences) for 5 minutes at room temperature. The sections were finally visualized using a confocal laser scanning microscope (FV1200, Olympus, Tokyo, Japan).

#### Cell culture and immunofluorescence studies

Immunofluorescence was performed by fixing C57BL/6 mouse cardiac fibroblasts (FBs) (Cat#M6300–57, Sciencell, CA, USA) with 4% PFA (Cat# 15710, Electron Microscopy Sciences, Hatfield, PA, USA) for 15 minutes and the cells were washed with PBS, permeabilized with 0.1% Triton X 100 (Cat# 1610407, Bio-Rad, Hercules, CA, USA) for 15 minutes, blocked with 4% BSA in PBS for 1 hour, and incubated overnight with an alpha-smooth muscle actin (α-SMA) antibody (Cat# NB300–978, Novus Biologicals, CO, USA) diluted (1:1000) in 1× PBS. The cells were again washed with 1× PBS, incubated with donkey anti-goat Alexa Fluor-488 (Cat# A11055; Invitrogen, Carlsbad, CA, USA) diluted (1:500) in 1× PBS for 90 minutes at room temperature, washed before being mounted with DAPI (Cat# LS-J1033–10; LifeSpan Biosciences, CA, USA) and imaged using a Zeiss confocal microscope (Carl Zeiss AG) at a magnification of 63×. Quantification was performed using ImageJ software (V1.54, NIH, Bethesda, MD, USA), and the results were plotted as corrected total cell fluorescence (CTCF). CTCF = Integrated density – (area of selected cell × mean fluorescence of background readings).

#### Cell lines, transfection, and coculture studies

Mouse HL-1 atrial cardiomyocytes were purchased from Sigma-Aldrich, St. Louis, MO, USA (Cat# SCC065). HL-1 cells were cultured in Claycomb media (Cat# 51800C, Sigma-Aldrich) containing 10% FBS, 0.1 mM norepinephrine (Cat# A0937, Sigma–Aldrich), 2 mM l-glutamine (Cat# G7513, Sigma) and penicillin/streptomycin (Cat# 15070063, Thermo Fisher Scientific, Waltham, MA, USA). The culture plates were precoated with gelatin/fibronectin overnight at 37°C before the cells were seeded. Mouse cardiac FBs were purchased from ScienCell (Cat# M6300–57, CA, USA) and cultured in FB medium-2 (Cat# 2331, ScienCell). The culture plates for FBs were precoated with poly-L-lysine (Cat# 0413, ScienCell) for 1 hour prior to seeding the cells. HL-1 cells were knocked down with small interfering RNA (siRNA) for Control scrambled (25 μM) (Cat# D-001320–10-05) and *Snrk* (Cat# J-051065–05-0005) (25 μM) from Horizon Discovery, Inc. (Waterbeach, UK). *Snrk* siRNA efficacy in HL-1 cells has been shown previously^7^ and was included in the present study. Briefly, HL-1 cells were seeded in 6-well plates approximately 24 hours prior to transfection. Multiple transfection protocols were tested, and Lipofectamine 2000 was determined to be optimal for HL-1 cell transfection. Compared with the other methods, the Lipofectamine 2000 reagent with 50% knockdown efficiency of the target SNRK resulted in the least amount of cell death. The transfection reagents were optimized to maintain the best cell viability and morphology. *Control* or *Snrk* siRNAs were transfected with Lipofectamine 2000 reagent (Cat# 11668030; Thermo Fisher Scientific) for 4–6 hours, changed to complete media and incubated for 48 hours until further experimentation, such as enzyme-linked immunosorbent assay (ELISA) or western blot was performed. For the coculture studies, *Snrk* was knocked down in HL-1 CMs that were then cocultured with cardiac FBs. Briefly, HL-1 CMs were grown on a 6-well transwell insert in a separate 6-well plate. *Snrk* (25 μM) and control (25 μM) siRNAs were transfected into HL-1 cells with lipofectamine for 4–6 hours, followed by a medium change to complete media. Then, the transwell inserts were placed on a 6-well plate that contained FBs and incubated for 48 hours under coculture conditions. A TGFβR inhibitor (SB 431542, Cat#1614, Tocris Bioscience, Bristol, UK) at a 1 µM concentration or Salmeterol xinofolate (negative control; Cat#S5068, Sigma) at 10 μM was added to cardiac FB wells for 24 hours before the cells were harvested for downstream analysis. Cardiac FBs were studied for protein expression via western blot and immunofluorescence.

#### Western blot assay

Proteins were extracted from CMs or human hearts using radioimmunoprecipitation assay (RIPA) buffer (Cat# R2078, Sigma) with a complete mini EDTA-free protease inhibitor cocktail (Cat# 11836170001, Roche, Basel, Switzerland) and PhosSTOP phosphatase inhibitor (Cat# 4906845001, Roche). After isolation, the total protein content was quantified. Cell lysates were probed with the following antibodies: Anti-rabbit SNRK (1:1000 dilution, Cat# GTX111380, Genetex), anti-rabbit TGF-β1 (1:500 dilution, Cat# GTX110630, Genetex), anti-goat α-smooth muscle actin (1:500 dilution, Cat# NB300–978, Novus Biologicals), anti-goat Smad 2/3 (1:200 dilution, Cat# AF3797, Novus Biologicals), anti-goat p-Smad 2/3 (1:1000 dilution, Cat# PA5–99378, Thermo Fisher Scientific), anti-rabbit TGFβR1 (1:1000 dilution, Cat# PA5–98192, Thermo Fisher Scientific), anti-rabbit p-TGFβR1 (1:1000 dilution, Cat# PA5–40298, Thermo Fisher Scientific), anti-mouse GAPDH (1:500 dilution, Cat# SC-47724, Santa Cruz Biotechnology, Santa Cruz, CA, USA) and anti-rabbit β-actin (1:1000 dilution, Cat# 4970, Cell Signaling Technology, Danvers, MA, USA). The respective primary antibodies were diluted in 1% Tris-buffered saline-Tween20 (TBS-T) and incubated overnight at 4°C in a shaker. The primary antibody-probed membranes were washed 3× with 1% TBS-T. Secondary HRP antibodies were diluted in 1% TBS-T for 1 hour at room temperature in a shaker and washed 3× with 1% TBS-T before the addition of chemiluminescence detection solution (Pierce^™^ ECL Western Blotting Substrate, Cat# 32209; Thermo Fisher Scientific). Anti-rabbit horseradish peroxidase (HRP) (1:1000 dilution, Cat# 7074, Cell Signaling Technology), anti-mouse horseradish peroxidase (HRP) (1:1000 dilution, Cat# 7076, Cell Signaling Technology), and anti-goat HRP (1:1000 dilution, Cat#205–052–176, Jackson ImmunoResearch Laboratories, West Grove, PA, USA) were secondary antibodies used for chemiluminescence detection (Pierce^™^ ECL Western Blotting Substrate, Cat#32209, Thermo Fisher Scientific). Quantification was performed via ImageJ software, and the results were plotted against the housekeeping control protein (β-actin) via GraphPad Prism software (V10.3, GraphPad Software, San Diego, CA, USA) as described previously.^7^

#### Enzyme-linked immunosorbent assay

Briefly, HL-1 CM cells were grown in a 30-mm dish, and the cells were transfected with *control* or *Snrk* siRNA (25 μM); 100 µL of the cell-free supernatant was used for the assessment of TGF-β1 protein levels. The protocol was followed according to the manufacturer’s instructions (Cat# DB100B, R & D Systems, MN, USA).

#### Statistical analysis

The data were analyzed via two-sample *t*-test, Welch’s *t*-test or analysis of variance (ANOVA) with Tukey’s test to adjust for multiple comparisons. The folded *F* test or Levene’s test was used to examine the homogeneity of the variance among different groups. Normality was evaluated by the Shapiro–Wilk test. When necessary for parametric assumptions, log transformation was employed. The Mann–Whitney–Wilcoxon test or Kruskal–Wallis test with Dwass, Steel, or the Critchlow–Fligner method for multiple comparison adjustment was used when parametric assumptions were not satisfied. *P* < 0.05 was considered statistically significant. SAS version 9.4 (SAS Institute Inc., Cary, NC, USA) was used for the statistical analyses.

## Results

### *Snrk* cardiac conditional knockout mice show more fibrosis in the atrial chamber compared with the ventricular chamber in the basal state

**Figure 1 |:**
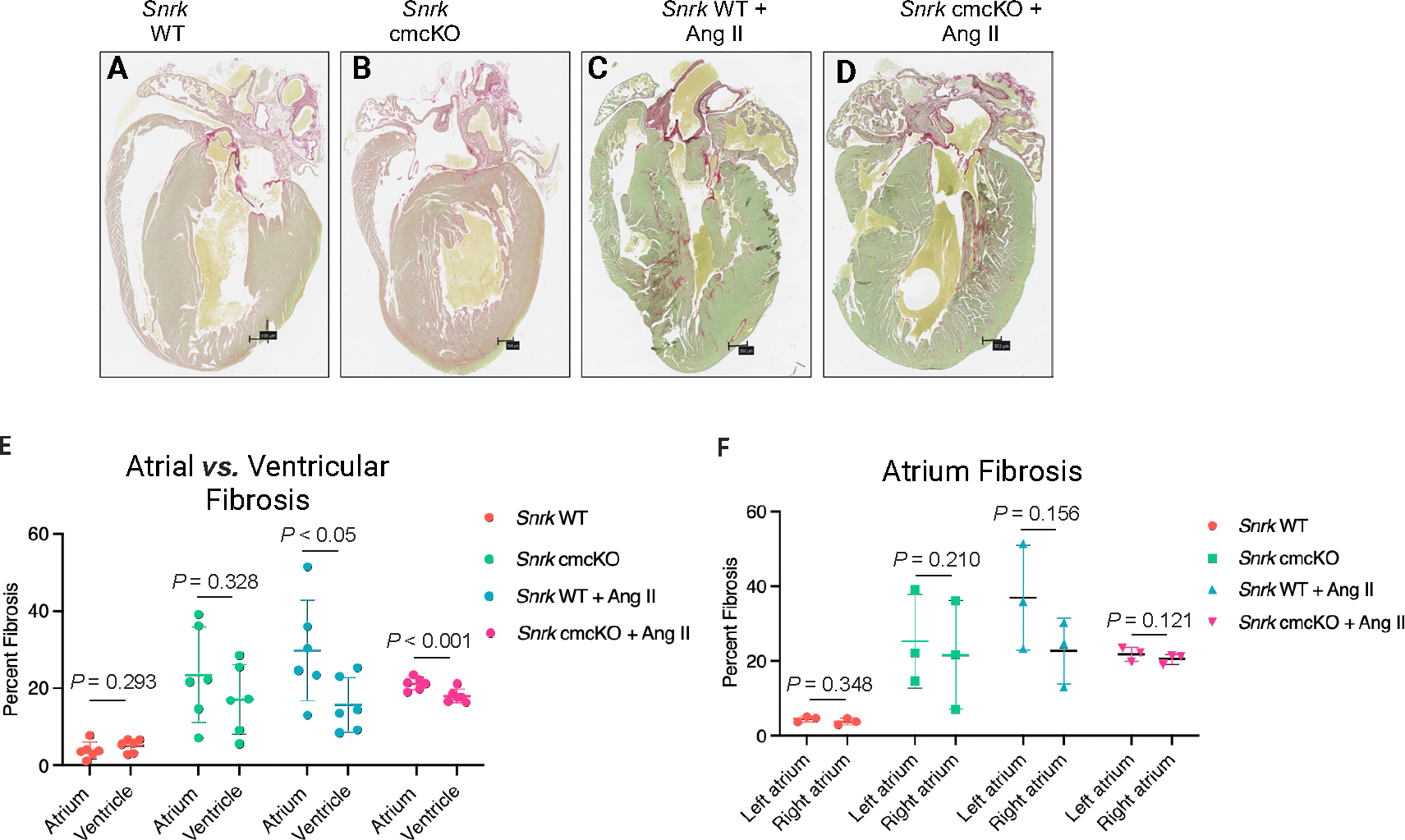
Chamber-specific effect of Snrk in cardiac conditional knockout (cmcKO) mice. The sample groups included the *Snrk* (WT), *Snrk* cmcKO, *Snrk* WT+ Ang II, and *Snrk* cmcKO + Ang II groups. *n* = 3 mice per sample group. The ages of the mice in the wild-type and *Snrk* cmcKO groups ranged from 3 months, 5 days (youngest), to 4 months, 18 days (oldest). (A–D) Sirius red-stained heart samples. Scale bars: 500 ± 4 μm for all images in the figure. (E) Quantification of atrial and ventricular chamber fibrosis across sample groups. For each mouse, we collected 2 data points per chamber. Thus, for *n* = 3 mice, we have 6 data points, as shown in E. (F) Quantification of left atrial and right atrial fibrosis across sample groups. All the data sets were normalized to body weight measurements. The results are presented as the mean ± SD for E and F. For E and F, analysis of variance was performed. Ang II: Angiotensin II; KO: knockout; WT: wild-type.

Previously, using a nonischemic Ang II-induced HF mouse model, we showed that SNRK acts as a cardiomyocyte-specific repressor of inflammation and fibrosis.^7^ When we evaluated fibrosis in a chamber-specific manner in *Snrk* cmcKO mice using Picrosirius red staining for collagen fibers, we observed a slightly higher fibrosis in the atrial chamber than in the ventricular chamber under both basal and Ang II-infused conditions (*P* < 0.05, *P* < 0.01; Figure 1A–E). Higher magnification section images of the Picrosirius red-stained collagen heart are provided (Additional Figure 2A and B). We also quantified fibrosis around the arteries, called perivascular fibrosis, and compared them to total tissue among all the groups (Additional Figure 2A–C). We observed a significant increase in perivascular fibrosis between the non-Ang II- and Ang II-treated groups (*P* < 0.05; Additional Figure 2C). These data are clinically relevant, as Ang II levels in the atria are increased in patients with atrial fibrillation and HF,^13^ and Ang II plays a key role in cardiac remodeling and dysfunction in the failing heart.^14,15^

We also compared the left atrium (LA) and right atrium (RA) for differences in fibrosis across the sample groups (Figure 1F). We observed a greater level of basal fibrosis in the *Snrk* cmcKO (LA: 25.33%; RA: 21.64%) group than in the *Snrk* WT (LA: 4.5%; RA: 3.78%) group. Upon Ang II infusion, the magnitude of change in the LA (8.2-fold) was greater in the *Snrk* WT Ang II (LA: 36.94%; RA: 22.66%) group than in the RA (5.9-fold) group. In contrast, the *Snrk* cmcKO Ang II (LA: 21.81%; RA: 20.51) (Figure 1F) group showed minimal changes over baseline *Snrk* cmcKO fibrosis levels. In addition, we evaluated the expression of α-SMA in mouse hearts and observed that the expression of α-SMA was greater in *Snrk* cmcKO, WT-Ang II, and *Snrk* cmcKO Ang II hearts than in WT hearts (Additional Figure 3A–C). We also investigated the infiltration of profibrotic Mac2 (Galectin3) macrophages (Additional Figure 4A–E) and did not observe an appreciable difference in their abundance in the atria vs. ventricle compartments of *Snrk* cmcKO hearts, although there was a total increase in macrophage infiltration in Ang II-infused and basal *Snrk* cmcKO hearts. These data collectively suggest that Snrk in cardiomyocytes serves a protective function to prevent fibrosis in the atrial and ventricular chambers of the heart.

### Higher Snrk expression in the atria is conserved in rats and humans and is associated with preventing atrial fibrosis and its related effects in heart failure

**Figure 2 |:**
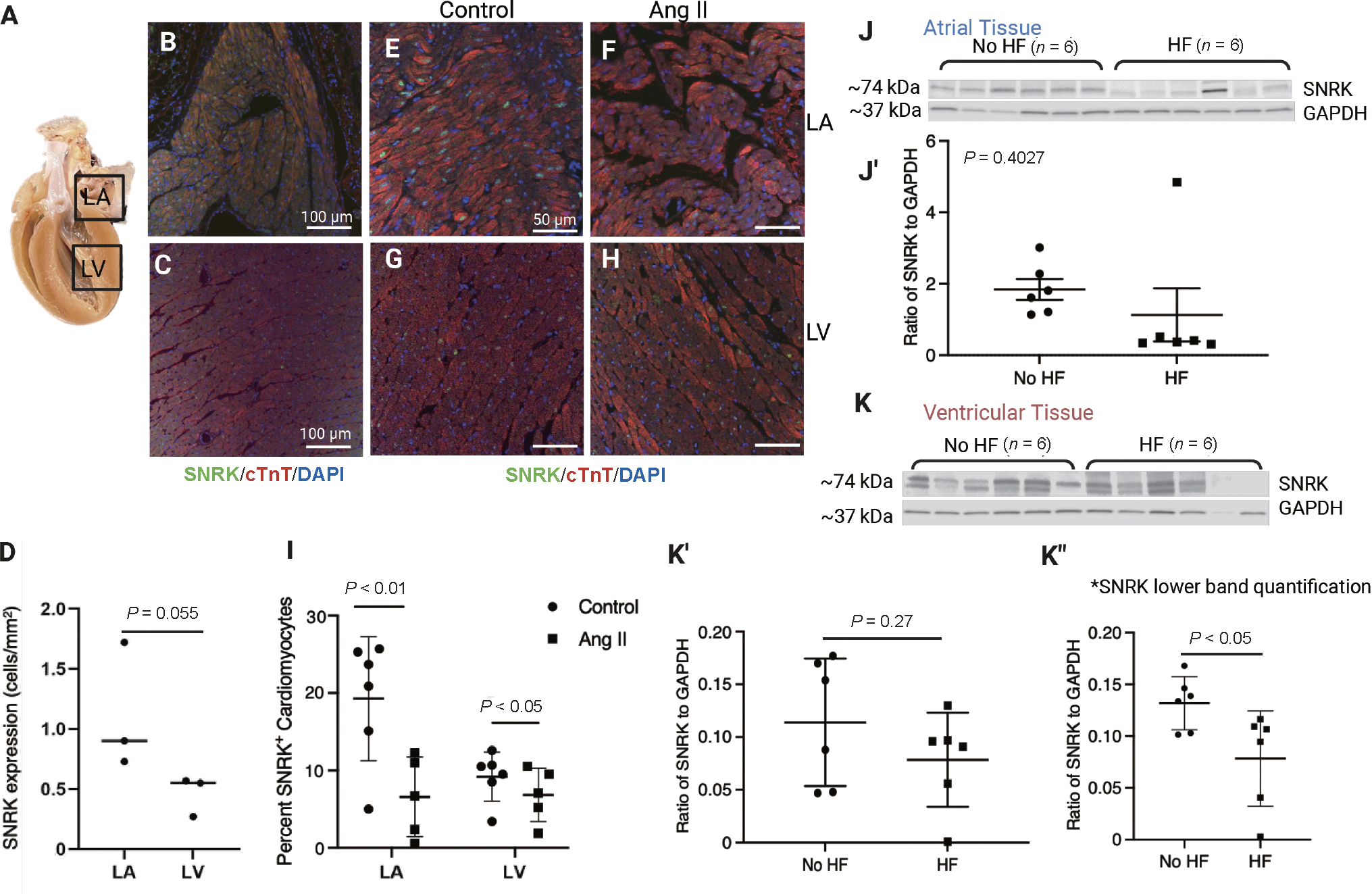
Chamber and septum-specific expression of SNRK levels. (A) The location of the rat heart where the images were taken for LA and LV. (B, C) Merged (SNRK, cTnT, and DAPI) immunofluorescence images of the septum-specific LA and LV. Scale bars: 100 μm. (D) Quantification of SNRK levels in the septum-specific LA vs. the LV. The results are presented as the mean ± SD. *n* = 3. (E–H) Immunofluorescence analysis of TnT costained with SNRK in septum-specific LA vs. LV cardiomyocytes treated with and without Ang II. Scale bars: 50 μm. (I) SNRK expression in cardiomyocytes in the LA and LV is shown. The results were quantified and are presented as the mean ± SD. *n* = 3. (J–K”) Atrial (J, J’) and ventricular (K, K’, K”) human heart failure and nonheart failure patient samples were analyzed for SNRK protein levels by immunoblotting. The SNRK lower protein band is marked with an asterisk (*) in the ventricular tissue and quantified in panel K”. The results were quantified and are presented as the mean ± SD. *n* = 6 per condition. Control blots for the housekeeping protein GAPDH were stripped and reprobed for SNRK. For I, analysis of variance was performed. For K”, a two-sample independent *t* test was performed. Ang II: Angiotensin II; cTnT: cardiac troponin; DAPI: 4’,6-diamidino-2-phenylindole; GAPDH: glyceraldehyde 3-phosphate dehydrogenase; HF: heart failure; LA: left atrium; LV: left ventricle.

To evaluate SNRK expression in atrial vs. ventricular cells in the heart, we performed immunofluorescence for SNRK in rat hearts (Figure 2A–C). Confocal immunofluorescence images of the SNRK protein in the atria and ventricles of a 56-day-old rat revealed higher SNRK expression in the LA than in the left ventricle (LV) (*P* = 0.055; Figure 2D). To determine SNRK expression in cardiomyocytes (CMs) in both chambers, we costained for cardiac troponin T (TnT) along with SNRK in the LA and LV (Figure 2E–H). Indeed, in control rats, the percentage of SNRK-positive CMs in the LA chamber was 21.4 ± 3.9%, whereas it was 10.1 ± 1.4% in the LV chamber (Figure 2I). Upon Ang II infusion, SNRK expression in LA CMs decreased to 6.3 ± 4.2% from 21.4% in controls, and in LV CMs, it decreased from 10.1% to 6.3% ± 3.1% (Figure 2I). Thus, more atrial CMs expressed SNRK, and upon Ang II infusion, the magnitude of change in SNRK expression was greater in LA CMs than in LV CMs.

To assess the relevance of SNRK expression in human tissue, heart tissue from six patients each (HF and no HF) undergoing ventricular assist device implantation or cardiac transplant procedures at Aurora, St. Luke Medical Center (Milwaukee, WI) was collected as part of an existing IRB at Advocate Aurora Health, Milwaukee, WI (Additional Figure 1). Compared with non-HF samples, HF samples were verified for increased CD68 macrophage staining and increased collagen staining for fibrosis (Additional Figure 1A–F). Atrial (Figure 2J, J’) and ventricular (Figure 2K, K’) lysates from patients with HF and without HF were generated and probed for the SNRK and GAPDH proteins. We observed that SNRK expression in atrial tissue was higher than that in ventricular tissue in non-HF patients (notice *y-axis* for non-HF samples, Figure 2J’–K’). Note that the SNRK antibody detected two bands in ventricular heart tissue compared with atrial tissue. The lower band (Figure 2K, black asterisk & 2K”) that matched the SNRK size (~74 kDa) and the upper band (Figure 2K’) in the ventricle samples were quantified. The lower band showed statistically significant differences across the HF and non-HF groups (Figure 2K”). Additionally, SNRK expression was lost in 5 out of the 6 HF atrial samples (Figure 2J). Taken together, the data from rats and humans suggest that SNRK expression is markedly higher in atrial CMs than in ventricle CMs and may have functional implications in preventing AF or HF associated with AF.

### SNRK in atrial cardiomyocytes represses transforming growth factor-β1 expression

**Figure 3 |:**
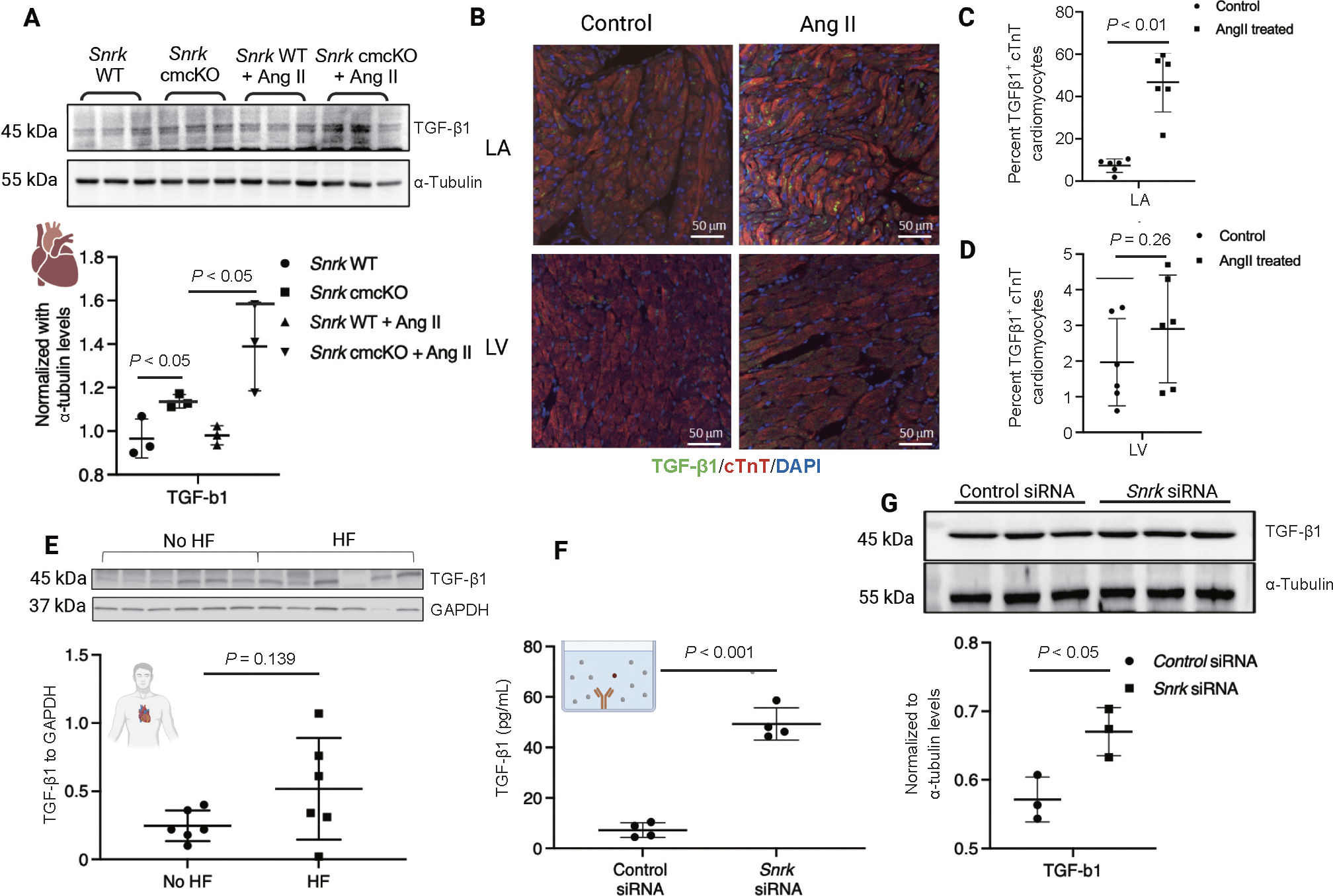
Cardiomyocyte SNRK represses TGF-β1 signaling. (A) TGF-β1 protein immunoblotting of whole heart tissue lysates from WT and *Snrk* cmcKO mice with and without angiotensin II (Ang II) treatment is shown. Quantification of TGF-β1 protein expression from the blot in panel A is shown. The results are presented as the means ± SDs. *n* = 3. For the control blots for the housekeeping gene, α-tubulin was stripped and reprobed for TGF-β1. (B) Immunofluorescence analysis of the expression of TGF-β1 in the septum-specific left atrium (LA) *vs*. LV cardiomyocytes with and without Ang II is shown. Scale bars: 50 µm. (C, D) Quantification of TGF-β1 in LA and LV rat cardiomyocytes is shown. The results are presented as the mean ± SD. *n* = 3. (E) Human HF and non-HF (No HF) atrial patient samples were analyzed for TGF-β1 protein levels by immunoblotting. The results are presented as the mean ± SD. *n* = 6. (F) *Snrk* was knocked down in mouse atrial HL-1 CMs, and the TGF-β1 protein levels in the cell-free supernatant were assessed via ELISA. The results are presented as the means ± SDs. *n* = 3. (G) *Snrk* was knocked down in mouse atrial HL-1 CMs, and TGF-β1 protein levels were assessed by immunoblotting. The results are presented as the mean ± SD. *n* = 3. Control blots for the housekeeping protein α-tubulin were stripped and reprobed for TGF-β1. For panels A, F, and G, a two-sample independent t test was performed. For C–E, Welch’s t test was performed. Ang II: angiotensin II; DAPI: 4′,6-diamidino-2-phenylindole; HF: heart failure; KO: knockout; LA: left atrium; LV: left ventricle; TGF: transforming growth factor; WT: wild-type

To investigate the underlying mechanism by which SNRK in CMs prevents fibrosis, we focused on TGF-β1 as a potential target of SNRK because TGF-β1 was previously implicated in promoting selective atrial fibrosis in a transgenic overexpressing mouse system.^9^ We investigated TGF-β1 protein levels in whole heart tissue lysates from vehicle- and Ang II-infused *Snrk* cmcKO mice (Figure 3A) and control rat hearts with and without Ang II (Figure 3B). TGF-b1 protein levels were higher in basal *Snrk* cmcKO hearts than in control hearts (Figure 3A), and upon Ang II treatment, these levels continued to rise even further (Figure 3B). In rats after Ang II infusion for 28 days, TGF-b1 was immunostained in rat atrial and ventricular CMs, and the results were quantified (Figure 3B–D). TGF-β1 expression was greater in Ang-II-infused LA CMs than in Ang-II-infused LV CMs (Figure 3C and D). In human atrial tissue, TGF-b1 protein levels also tended to be higher in HF than in non-HF patient samples (Figure 3E). Because the TGF-β1 protein is secreted, we probed for the TGF-b1 protein via ELISA in control and efficacy-confirmed *Snrk* siRNA-knockdown HL-1 adult immortalized mouse atrial cell supernatants (Figure 3F). We detected 48 pg/mL of TGF-β1 in the *Snrk* siRNA group compared with the 7 pg/mL control siRNA group. The increased TGF-β1 protein levels detected by ELISA were also confirmed by western blots of *Snrk* siRNA HL-1 cell lysates (Figure 3G). These expression studies collectively suggest that SNRK regulates TGF-b1 expression in atrial CMs.

### Transforming growth factor-β1 released from SNRK-knockdown atrial cardiomyocytes promotes fibrosis

**Figure 4 |:**
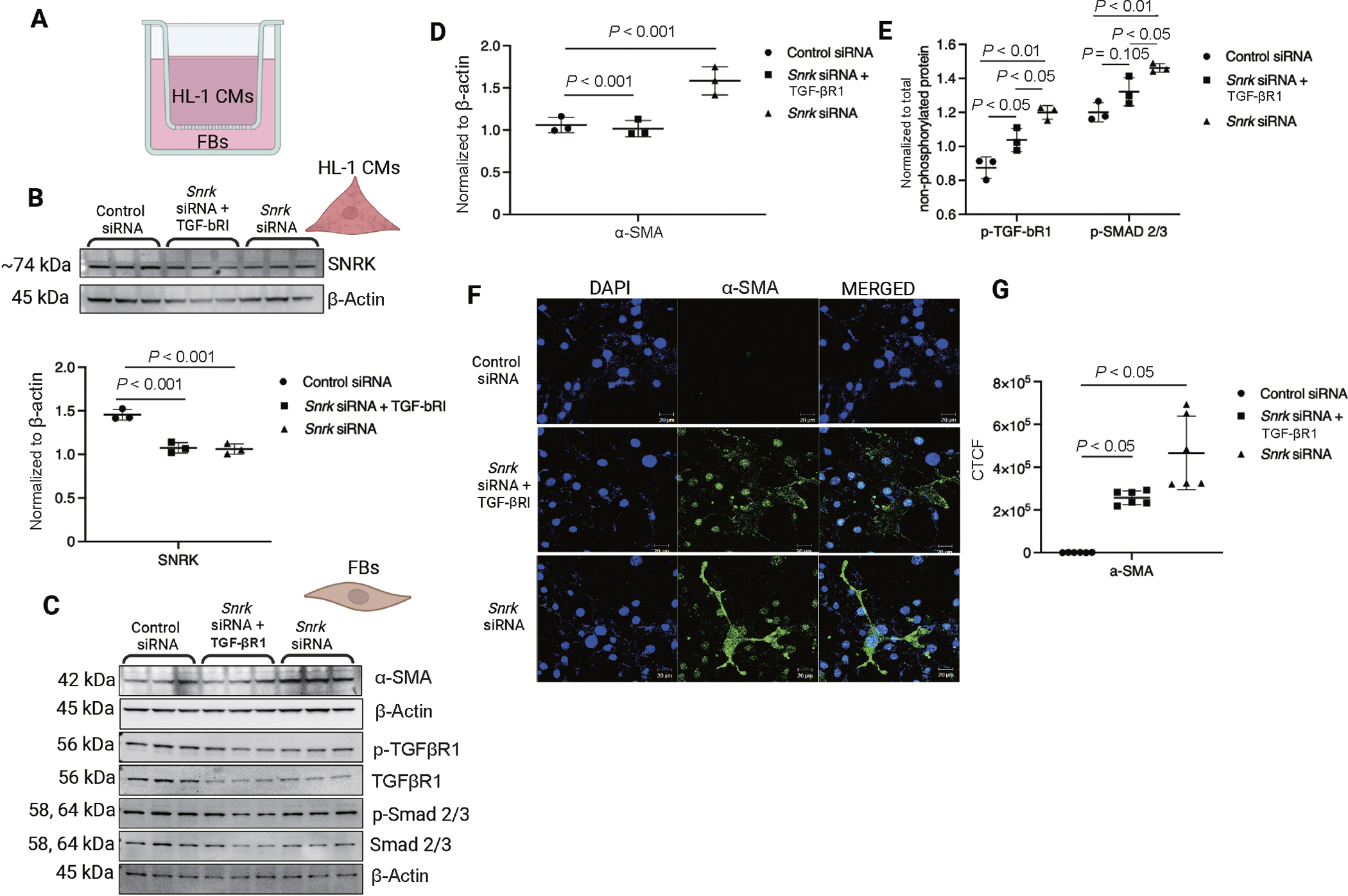
CM-mediated SNRK regulates TGF-β1 for fibroblast activation. (A) The transwell coculture system is depicted with mouse atrial HL-1 CMs inside the insert and mouse cardiac FBs in the bottom of the well. (B) *Snrk* was knocked down in mouse atrial HL-1 CMs, and the knockdown efficiency was assessed. Western blot of mouse atrial HL-1 CM with *Snrk* knockdown is shown along with the quantification below. The results are presented as the means ± SDs. *n* = 3. (C) Cardiac fibroblasts were assessed for the expression of p-TGFβR1, p-Smad 2/3 and α-SMA by immunoblotting and quantified. The experiments were performed with and without a TGFβR inhibitor (TGF-βRI) (1 µM) on cardiac fibroblasts. (D, E) Quantification of α-SMA p-TGFβR1 and p-Smad 2/3 protein levels are presented as the means ± SDs. *n* = 3. Control blots for the housekeeping protein β-actin were stripped and reprobed for α-SMA p-TGFβR1, p-Smad 2/3, TGFβR1, and SMAD 2/3. (F) Mouse cardiac fibroblasts (cocultured with *Snrk*-knockdown atrial HL-1 CMs) were assessed for the expression of α-SMA by immunofluorescence. Scale bars: 20 µm. (G) Quantification was performed in ImageJ to calculate the corrected total cell fluorescence (CTCF). CTCF = Integrated density – (area of selected cell × mean fluorescence of background readings). Two random areas per slide per condition were quantified. The results are presented as the mean ± SD. *n* = 3. Control blots for the housekeeping protein β-actin were stripped and reprobed for SNRK. For B–E, analysis of variance with Tukey’s adjustment test was performed. For G, the Kruskal–Wallis test with Dwass, Steel, and Critchlow-Fligner methods was used. CMs: Cardiomyocytes; DAPI: 4’,6-diamidino-2-phenylindole; TGF: transforming growth factor; α-SMA: alpha-smooth muscle actin

TGF-β1 is a well-studied molecule that plays a critical role in cardiac fibrosis events.^9,11,16,17^ To investigate the direct role of TGF-β1 secreted from *Snrk-*knockdown atrial CMs in cardiac fibrosis, using a transwell insert system, we performed coculture studies with HL-1 atrial CMs cultured in the well insert and mouse cardiac FBs from commercial sources in the bottom well (Figure 4A). HL-1 atrial CMs were subjected to *Snrk* knockdown, and the loss of the SNRK protein was investigated via western blot assay (Figure 4B). *Snrk* knockdown CMs and control siRNA CMs were cocultured with mouse cardiac FBs treated with a TGFβR inhibitor (TGF-βRI) or the negative control Salmeterol xinafoate, a non-TGF-β signaling pathway inhibitor (beta-2 adrenergic receptor agonist drug). FB lysates were subjected to western blots for select proteins in the TGF-β1 signaling pathway, including phosphor-TGF-beta receptor 1 (p-TGF-βR1), total TGF-β1, phospho-Smad-2 and -3 (p-Smad2/3). For FB activation in coculture, the alpha-smooth muscle actin (α-SMA) protein was investigated. The Western blot results of FBs cultured with *Snrk*-knockdown atrial CMs revealed increased protein expression of the TGF-β1 signaling pathway (p-TGFβR1 and Smad2/3 proteins) (Figure 4C) and α-SMA (Figure 4D and E). FBs treated with p-TGF-bRI and cocultured with *Snrk*-knockdown CMs presented a significant decrease in the expression of all three proteins tested (Figure 4C–E). We also performed immunofluorescence on cocultured FBs, which revealed that those cultured with *Snrk*-knockdown CMs showed a significant increase in the activation of α-SMA in FBs (Figure 4F and G) than those in the presence of TGF-βR1. The specificity of α-SMA staining was demonstrated by negative staining in Salmeterol xinafoate control FBs (Additional Figure 5). Collectively, the results of the coculture experiments suggest that SNRK-associated TGF-β1 secreted from CMs activates the TGF-β1 signaling pathway on FBs, leading to a myofibroblast and fibrotic phenotype.

## Discussion

The salient features of this study include increased SNRK expression in the atrial chamber compared with ventricles in the mammalian heart, the sensitivity of atrial fibrosis to declining cardiac function, and the identification of SNRK atrial cardiomyocyte-driven repression of TGF-β1 signaling to control cardiac fibrosis, a key cardiac repair process during cardiac regeneration post HF.

Immunofluorescence staining of the SNRK protein in mammalian hearts revealed that SNRK is expressed at one-fold higher levels in the LA than in the LV under normal baseline conditions. Specifically, in CMs, this trend continues, with SNRK protein expression being one-fold higher in left atrial CMs than in left ventricular CMs. The increased atrial SNRK protein expression is also recapitulated in human hearts. SNRK protein expression is significantly elevated in the atrium compared with the ventricle in non-HF samples. In addition, compared with that in non-HF samples, SNRK expression is decreased in HF patients. These findings collectively suggest that SNRK expression in the LA is responsible for repressing fibrosis. A recent single-cell analysis of human heart tissue revealed that the atrium comprises 30.1% CMs and 24.3% FBs, whereas 49.2% CMs and 15.5% FBs are present in the ventricle.^18^ Therefore, the presence of more FBs in the atria may increase the propensity for fibrosis in the atrium.

In addition to the atrial-specific expression of SNRK, analysis of fibrosis revealed that in the basal state, *Snrk* cmcKO hearts presented a slightly higher level of fibrosis in the atria than in the ventricles. This trend continued in the Ang II-infused mice, albeit in an exaggerated manner. When fibrosis was compared between the LA and the RA in *Snrk* WT mice, due to minimal changes were observed. Compared with the RA group, the LA group presented more fibrosis upon Ang II infusion, but this difference was not statistically significant. Compared with those of the *Snrk* WT mice, both the LA and the RA of the *Snrk* cmcKO mice presented increased basal fibrosis. Upon Ang II infusion, LA and RA fibrosis were not different between the *Snrk* cmcKO state and the basal state. It has been revealed that *Snrk* cmcKO mice die at 9 months.^6^ In the present study, upon Ang II infusion into 4–5-month-old *Snrk* cmcKO mice, they died within 14 days. Taken together, our data suggest that a heart with increased fibrosis levels in the atria has decreased cardiac function and is increasingly susceptible to HF. Clinically, atrial fibrosis has been proposed as a substrate for promoting atrial fibrillation, a common clinical arrhythmia associated with HF,^19,20^ and our preclinical studies here support this proposition.

The SNRK protein expression data revealed increased atrial expression, especially in the LA. However, fibrosis was observed in both the LA and the RA in *Snrk* cmcKO mice in the basal state. These observations suggest that mechanisms for fibrosis are connected within the left and right sides of the heart and across the atrial and ventricular chambers. However, whether FBs of the atrium vs. ventricle or left vs. right sides of the heart mediate selective crosstalk mechanisms with CMs is not known and remains to be determined.

Given the underlying mechanisms that mediate atrial cardiomyocyte SNRK-driven repression of fibrosis, we focused on TGF-β1. Previous work showed that the overexpression of a mutant (C33S) form of constitutively active TGF-β1 expression under a cardiac-restricted *α-myosin heavy chain* promoter resulted in selective atrial chamber-specific fibrosis but not ventricular chamber fibrosis even though the protein was expressed constitutively in both chambers.^9^ The underlying mechanism for this selective atrial fibrosis in these mutant TGF-β transgenic overexpressing mice is not known. Our data provide one possible molecular explanation for this observation. Data from mouse, rat, and human hearts and *in vitro* mouse atrial CMs revealed that a loss or decrease in CM SNRK protein levels increased the expression of TGF-β1, suggesting that SNRK acts as a regulator of TGF-β1 expression. Exactly how this occurs is not known. Based on the results here, we propose that TGF-β1 levels in atrial CMs are controlled by SNRK. Thus, the loss of SNRK in atrial CMs results in the release of more TGF-β1 into the supernatant, which then acts on the cognate receptor (TGFβR1) in cardiac FBs to activate the downstream Smad pathway to promote fibrosis. The increase in phosphorylated TGFβR1 and Smad 2/3 protein levels in cardiac FBs cocultured with *Snrk-*knockdown HL-1 atrial CMs suggests that elevated TGF-β1 levels in atrial CMs can trigger TGF-β1 signaling in FBs. Furthermore, the high levels of SMA in cardiac FBs cocultured with *Snrk-*knockdown HL-1 atrial CMs confirm the activation of FBs to myofibroblasts. These results collectively support the hypothesis that SNRK-mediated TGF-β1 secretion from CMs is sufficient to induce FB activation to myofibroblasts through a specific receptor-initiated signaling. The activation of cardiac FBs to myofibroblasts is one of the key processes in cardiac fibrosis and is commonly associated with most cardiovascular diseases.^21^ The activation of the Smad pathway in myofibroblasts results in the recruitment and activation of intracellular effectors to send signals to the nucleus, which triggers gene transcription of matrix components such as α-SMA to promote fibrosis.^22^ Thus, we conclude that SNRK-associated TGF-β1 atrial CM signals through TGFβR promote myofibroblast and fibrotic phenotypes, processes directly linked to cardiac repair and regeneration after HF.

### Limitations

Our study has some limitations, as we deleted *Snrk* from all CMs. Thus, we cannot eliminate the contribution of ventricular CMs to fibrosis. An atrial-specific CRE excision of SNRK is necessary to bolster our conclusions. Mac2^+^ macrophage infiltration does not have any chamber-specific effect other than a basal level increase in the *Snrk* cmcKO- and Ang II-treated mice compared with the WT mice. Furthermore, in-depth analysis of multiple macrophage markers is needed to reach conclusions. Finally, our numbers are low, and higher numbers offer more granularity to the chamber and side-specific effects of cardiomyocytes on fibrosis.

## Conclusion

We identified that SNRK is more highly expressed in the atria compared with the ventricles of multiple species and regulates the myofibroblast driver TGF-β1 to protect the atrium and prevent fibrosis. This chamber-specific SNRK-TGF-β1 axis may be highly beneficial for developing targeted approaches to prevent atrial fibrosis in nonischemic HF. We conclude that the metabolic enzyme SNRK in atrial cardiomyocytes regulates the expression of the key myofibroblast driving factor TGF-β1 during the cardiac fibrosis repair process in nonischemic HF.

## Supplementary Material

Supplemental figures **Additional Figure 1:** Human heart failure and non-heart failure samples assessed for inflammation and fibrosis.
**Additional Figure 2:** Representative images of the whole heart section for Picrosirius Red fibrosis staining.
**Additional Figure 3:** Mouse heart tissue section assessed for the expression of alpha-smooth muscle actin.
**Additional Figure 4:** Mouse heart tissue section assessed for Mac2 macrophages.
**Additional Figure 5:** Salmeterol xinafoate negative control for SMA activation.

## Data Availability

All relevant data are within the paper and its Additional files.
