# Supplementary material for: SNRK facilitates cardiac repair associated with nonischemic fibrosis: regulating transforming growth factor-beta1 levels in atrial cardiomyocytes
Source: Regen Med Rep. Author manuscript; Available in PMC 2025 Jun 27. (PMC12204380; doi:10.4103/regenmed.regenmed-d-25-00009)
Supplement: Supplemental figures — Additional Figure 1: Human heart failure and non-heart failure samples assessed for inflammation and fibrosis. Additional Figure 2: Representative images of the whole heart section for Picrosirius Red fibrosis staining. Additional Figure 3: Mouse heart tissue section assessed for the expression of alpha-smooth muscle actin. Additional Figure 4: Mouse heart tissue section assessed for Mac2 macrophages. Additional Figure 5: Salmeterol xinafoate negative control for SMA activation. [file NIHMS2088512-supplement-Supplemental_figures.pdf]

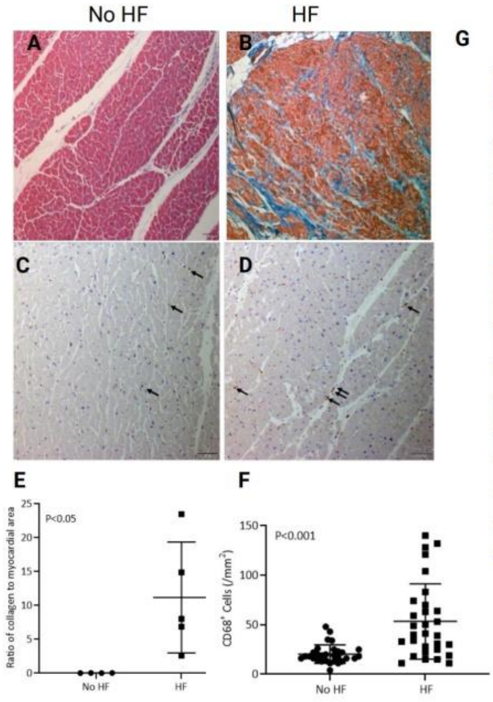

G

Sample ID	Sample type	Disease/underlying conditions	Age (Y)	Gender	HFpEF/HFrEF
**Heart Failure Samples**					
ND-1	Donor Dead	HF/Diabetes/Afib	81	M	N/A
HT23	Heart Transplant	HF/Cancer	62	M	HFrEF
HT24	Heart Transplant	HF/Diabetes	67	M	HFrEF
HT25	Heart Transplant	HF/Diabetes	32	F	HFrEF
HT26	Heart Transplant	HF/Diabetes	42	M	HFrEF
HT29	Heart Transplant	HF/Cancer	64	M	HFrEF
**Non-Heart Failure Samples**					
ND-2	Donor Dead	Hyperlipidemia/COPD	78	F	N/A
ND-3	Donor Dead	Hyperlipidemia/GERD/Neuropathy	50	F	N/A
ND-5	Donor Dead	Renal Failure	45	M	N/A
ND-7	Donor Dead	Diabetes/Hyperlipidemia	80	F	N/A
ND-8	Donor Dead	Hyperlipidemia/GERD	64	M	N/A
ND-9	Donor Dead	Alzheimer	81	F	N/A

Additional Figure 1 Human heart failure and non-heart failure samples assessed for inflammation and fibrosis.

(A, B, E) HF and no HF samples were stained for collagen to assess fibrosis (A, B) and quantified with the ratio of collagen-positive area to myocardial area (E). (C, D, F) HF and no-HF samples were stained for CD68-positive macrophages (C, D) and quantified per mm^2 (F). (G) Representative table of human patient sampling data. For E and F, a two-sample independent *t*-test was performed. HF: Heart failure.

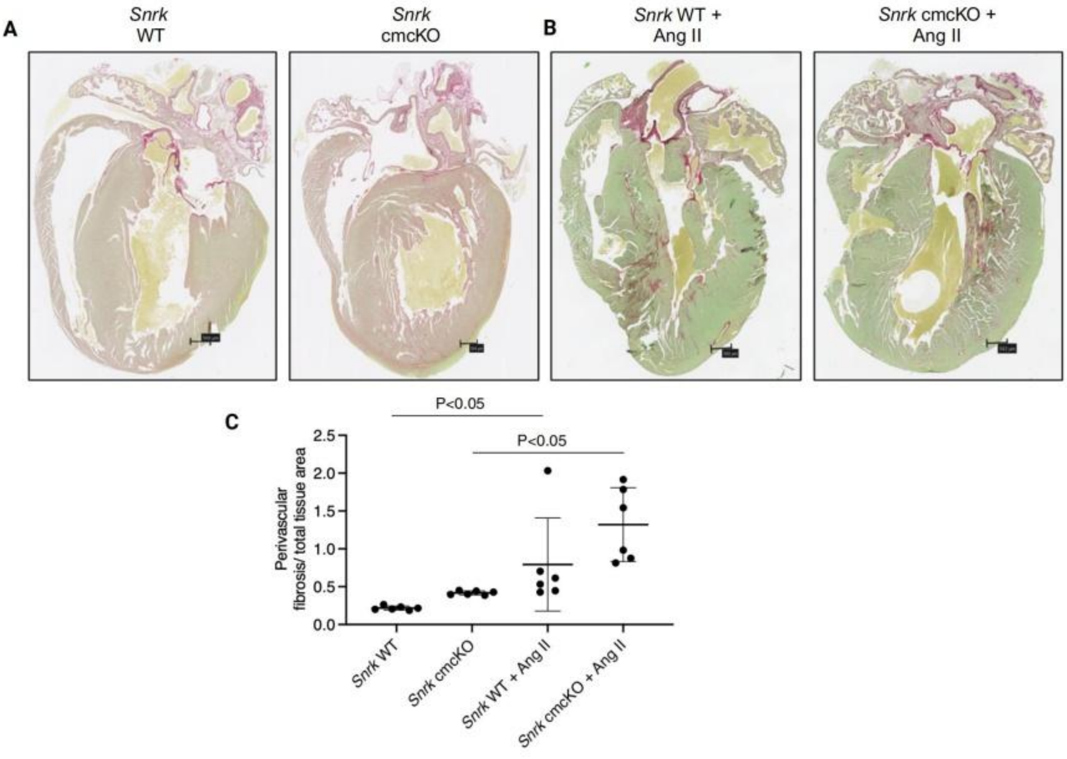

Additional Figure 2 Representative images of the whole heart section for Picrosirius Red fibrosis staining. (A) *Snrk* WT and *Snrk* cmcKO whole heart section. (B) *Snrk* WT + Ang II and *Snrk* cmcKO + Ang II whole heart section. Scale bars: 500 ± 4 µm. (C) Whole heart tissue samples were quantified for perivascular fibrosis with the total tissue area. Results were presented as mean ± SD. *n* = 3. Mann-Whitney-Wilcoxon statistical test was done. Ang II: Angiotensin II; KO: knockout; WT: wild-type.

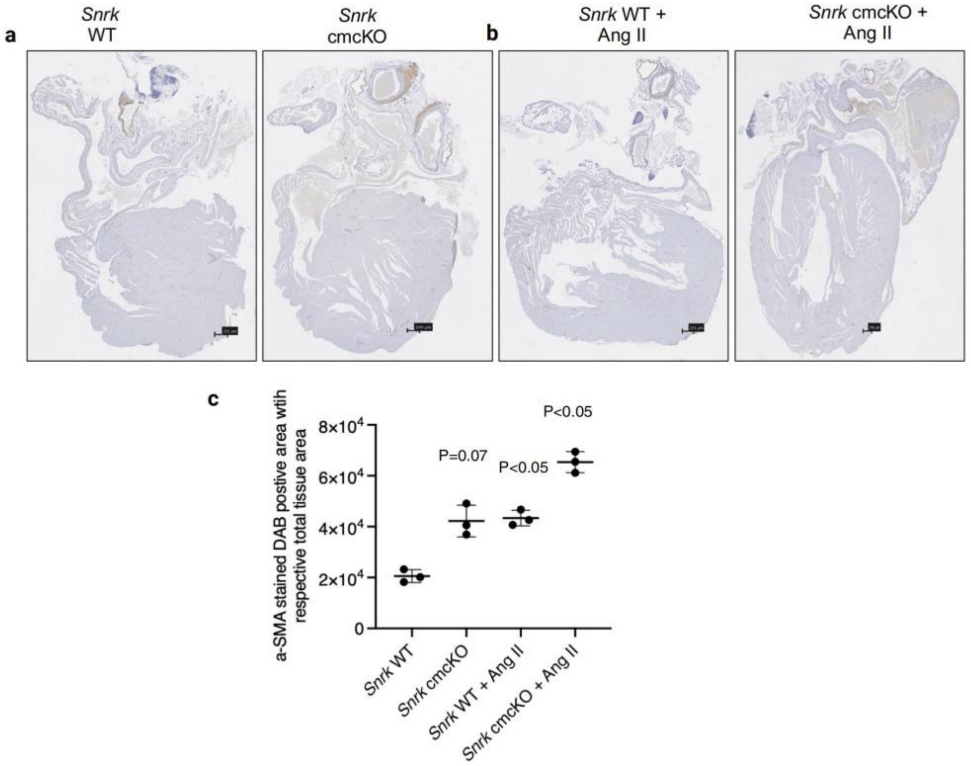

Additional Figure 3 Mouse heart tissue section assessed for the expression of alpha-smooth muscle actin.
(A-C) *Snrk* WT, *Snrk* cmcKO, *Snrk* WT + Ang II, and *Snrk* cmcKO + Ang II tissue sections stained for α-SMA (A, B) and quantified (C). Results are presented as mean ± SD. n=3. Statistical analysis (*P* value) was compared with WT. Scale bars: 250±3 μm. Mann-Whitney-Wilcoxon statistical test was performed. Ang II: Angiotensin II; KO: knockout; WT: wild-type.

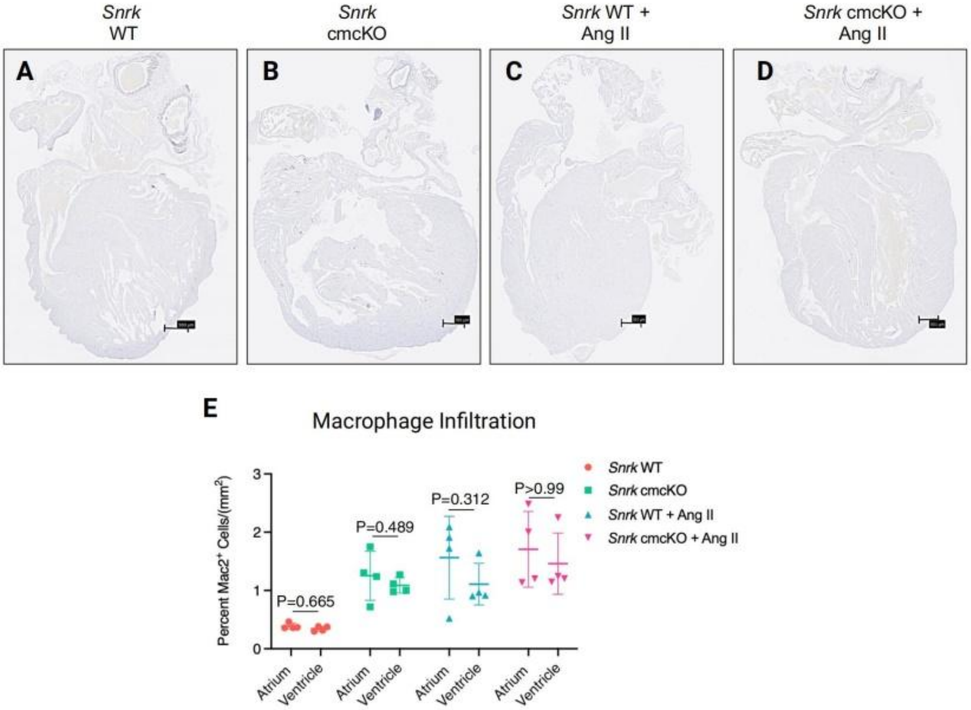

Additional Figure 4 Mouse heart tissue section assessed for Mac2 macrophages.

(A-D) *Snrk* WT, *Snrk* cmcKO, *Snrk* WT + Ang II, and *Snrk* cmcKO + Ang II tissue sections stained for $Mac2^+$ atrial and ventricular chambers and quantified (E). Results are presented as mean ± SD. $n = 3$. Statistical analysis (*P* value) was compared with WT. Scale bars: 500±1 μm. Mann-Whitney-Wilcoxon statistical test was performed. Ang II: Angiotensin II; KO: knockout; WT: wild-type.

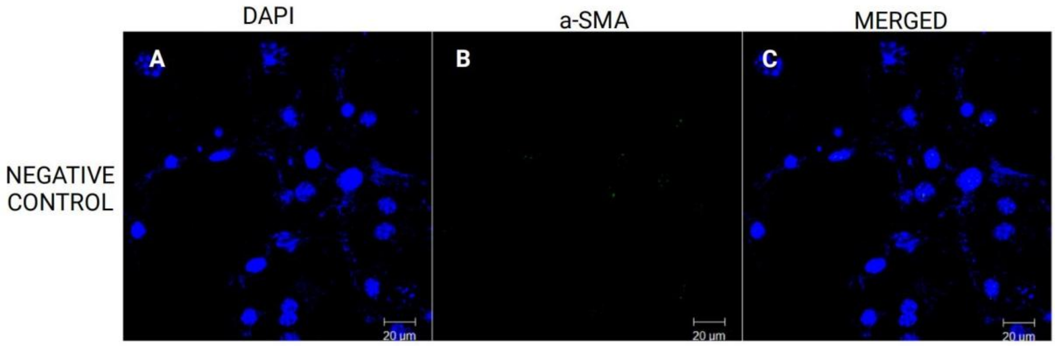

Additional Figure 5 Salmeterol xinafoate negative control for SMA activation.
(A-C) Salmeterol xinafoate (10 μM) treated mouse cardiac fibroblasts were assessed for DAPI (A) α-SMA (B) staining in fibroblasts. The results show that salmeterol xinafoate does not activate SMA, and is considered a negative control for the co-culture inhibitor experiment. Scale bars: 20 μm. DAPI: 4',6-Diamidino-2-phenylindole; α-SMA: alpha-smooth muscle actin.
